# Processed food consumption and risk of non-communicable diseases (NCDs) in South Africa: evidence from Demographic and Health Survey (DHS) VII

**DOI:** 10.1017/jns.2024.13

**Published:** 2024-03-27

**Authors:** Swapnil Godbharle, Hema Kesa, Angeline Jeyakumar

**Affiliations:** 1 Food Evolution Research Laboratory (FERL), School of Tourism and Hospitality, College of Business and Economics, University of Johannesburg, Johannesburg, South Africa; 2 Department of Health Sciences, Savitribai Phule Pune University, Pune, India; 3 Department of Nutrition, University of Nevada, Reno, Nevada, USA

**Keywords:** Dietary patterns, Non-communicable diseases, Processed food, South Africa

## Abstract

We aimed to analyse the association between processed food consumption and the risk of non-communicable diseases (NCDs) in South Africa. In this empirical study, we analysed nationally representative secondary data obtained from the South African Demographic and Health Survey (SADHS) VII. The survey included 13,288 occupied households, of which 11,083 were interviewed. In the interviewed households, 12,717 eligible adults aged 15 and older were identified and 10,336 were successfully interviewed. The study included four processed food groups (i.e. fried foods, takeaway foods/fast foods, salty snacks/packed chips, and processed meats) and eight NCDs (i.e. hypertension, cardiac arrest, cancer, stroke, hypercholesterolaemia, diabetes, chronic bronchitis, and asthma). As per the logistic regression results following adjustment, none of the disease states showed association with all four processed food groups. However, at least three processed food groups showed a significant positive association with hypertension, cardiac arrest, and diabetes. Two processed food groups showed significant positive association with stroke, and chronic bronchitis; one with hypercholesterolaemia and asthma; and cancer was not associated with any food groups. Processed meat and salted snacks/packed chips were each associated with five chronic conditions. In summary, we found that the consumption of any of the processed food groups increased the risk of NCDs in the South African population. Enabling policy and regulatory efforts in the production and distribution of processed foods, combined with improved awareness among the population need to be prioritised for immediate action. Facilitating the populations to choose traditional healthy diets would be a sustainable strategy for the prevention of NCDs.

## Introduction

Changes in dietary behaviour have significantly contributed to the demand for processed foods, leaving behind traditional healthy diets. Globally, modern diets have been associated with non-communicable diseases (NCDs).^(1)^ The global burden of disease (GBD), 2017 estimated 11 million deaths and 255 million disability-adjusted life years (DALYs) attributable to dietary risk factors.^(2)^ Certain dietary constituents that contribute to ill health such as trans fats and sodium/salt in processed foods have been a rising concern that the 13th general programme of work, World Health Organisation (WHO),^(3)^ has identified these as priority areas to promote health and wellbeing of populations. The GBD 2017 estimated that high sodium intake was associated with 3 million deaths and 70 million DALYs; low intake of whole grains with 3 million deaths and 82 million DALYs, and low intake of fruits with 2 million deaths and 65 million DALYs.^(2)^

Irrespective of the development status, all countries show a change in dietary behaviours. However, the trends and contributing factors differ between countries. While developed countries witnessed an increase in dining at full-service restaurants,^(4)^ fast-food outlets increased in developing countries.^(5–7)^ As in other developing countries, in Africa too, the shift in intake of processed foods is influenced by personal, cultural, environmental, and economic factors. Within Africa, unhealthy dietary choices varied between age, gender, socio-economic class, and ethnicity.^(8,9)^ Increased supply sustained by the expanding food processing sector, to meet the growing demand, adds to the changing trends.^(10)^ As a consequence, a rise in the burden of non-communicable diseases in Africa has been documented.^(11)^ Paradoxically, a recent meta-analysis emphasised food insecurity as a risk factor for metabolic disorders, which highlighted the causal pathway between diet-sensitive NCDs in Africa.^(12)^ The WHOs’ NCD progress monitor reported 50% and 88 % of deaths due to NCDs in seven small countries in Africa, indicating unsustainable urbanisation, lifestyle changes, and the multiple burdens of diseases.^(13,14)^

In South Africa, deaths due to major NCDs such as cardiovascular diseases, cancer, diabetes, and chronic lower respiratory diseases increased by 58.7% over 20 years, from 103,428 in 1997 to 164,205 in 2018.^(15)^ This increase in deaths due to NCDs in South Africa has been linked to four lifestyle risk factors of unhealthy diet, physical inactivity, tobacco use and inappropriate use of alcohol.^(16)^ Among these, the prevalence of diet-related NCDs is disturbingly high, accounting for 51% of the country’s annual deaths.^(17)^ In the past few years, the consumption of processed and ultra-processed food and beverages has also increased in South Africa.^(18)^ The increasing consumption of unhealthy products has been made possible by the wide availability, affordability, palatability, and convenience of unhealthy processed foods. The non-availability of healthier choices, and less time for preparation, has also resulted in unhealthy food choices among low-income communities in South Africa.^(9)^ Similarly, limited empirical evidence on the emerging diet patterns specifically from South Africa, a country experiencing rapid urbanisation, calls for utilising national surveys to study associations.

The present work, therefore, analysed the association between processed food consumption and the risk of NCDs in South Africa using the South African Demographic and Health Survey (SADHS) VII (2016).

## Methods

### Data

The DHS is a nationally representative household survey conducted in more than 80 countries since 1984. This project is primarily funded by the United States Agency for International Development, which has conducted over 230 household surveys across nations. The data collected includes a wide range of topics: (a) reproductive health viz fertility, contraception, sexual activity, (b) maternal and child health viz nutrition, child mortality, and (c) infections viz exposure to the risk of HIV, malaria, and other miscellaneous variables. Additionally, clinical and nutritional parameters such as anthropometry, anaemia, hypertension, and HbA1c levels are also measured. The DHS provides vital information to inform policy and to monitor and evaluate the country’s public health progress.

Statistics South Africa (Stats SA), in partnership with the South African Medical Research Council, conducted the SADHS 2016. Data were analysed from participants aged 15 years or older from the seventh round of SADHS in 2016. The sampling frame used for the SADHS 2016 is the Statistics South Africa Master Sample Frame (MSF), which was created using Census 2011 enumeration areas (EAs). In the MSF, EAs of manageable size were treated as primary sampling units (PSUs), whereas small neighbouring EAs were pooled together to form new PSUs, and large EAs were split into conceptual PSUs. The frame consisted of information about the geographic type (urban, traditional, or farm) and the estimated number of residential dwelling units (DUs) in each PSU.

The SADHS 2016 followed a stratified two-stage sample design with a probability proportional to the size sampling of PSUs at the first stage and systematic sampling of DUs at the second stage. A total of 750 PSUs were selected from the 26 sampling strata that yielding 468 selected PSUs in urban areas, 224 PSUs in traditional areas, and 58 PSUs in farm areas. A total of 15,292 households were selected for the sample, of which 13,288 were occupied. Of the occupied households, 11,083 were successfully interviewed. In the interviewed households, 12,717 eligible adults aged 15 and older were identified and 10,336 were successfully interviewed with the adult health module, which yielded a response rate of 81%.^(19)^ Individuals who either declined participation or were not present during the time of the interview, as well as interviews with incomplete responses, were excluded from the final sample. As this was an empirical study that used secondary data, it was exempt from the Institutional Review Board approval as per the Institutional Ethics Committee guidelines.

### Measures

The present study had eight common chronic conditions (hypertension, cardiac arrest, cancer, stroke, hypercholesterolaemia, diabetes, chronic bronchitis, and asthma) as the main outcome variable. These eight common chronic conditions were included in survey questionnaire for data collection. The participants were asked if a doctor, nurse, or other health worker had communicated to them that they have or have had any of the eight common chronic conditions. The survey instrument also collected participants’ information on socio-demographic and economic characteristics such as age, education, place of residence, marital status, employment, and wealth status. It also elicited information regarding the four processed food groups that included (1) fried foods (hot chips, fried fish, fried chicken, fried meat, vetkoek or doughnuts), (2) takeaway foods or fast foods (from places like Chicken Licken, KFC, Captain DoRego’s, Steers, Nando’s, McDonald’s, or pizza delivery), (3) salty snacks or packed chips (such as Doritos, cheese curls, salted nuts, or salty biscuits), and (4) processed meats (such as polony (a large sausage made from a mixture of beef and pork), viennas, meat pies, or sausage rolls). The participants were asked, ‘how often they consumed fried foods/fast foods or takeaway foods/packed chips or salty snacks/processed meats?’ The responses to the questions were ‘never’, ‘occasionally’, ‘at least once a week’, or ‘every day’. For our analysis, the above four food categories have been grouped under processed foods. As per the United States Department of Agriculture processed foods are defined as those that have undergone any change to their natural state viz., cutting, chopping, cleaning, cooking, canning, dehydrating, drying, freezing, milling, mixing, packaging, and washing.^(20)^

### Analytic approach

Cross-tabulations and summary statistics were performed to describe the study population. Simple frequencies, summary measures, tables, and figures were used to present the data. Age was analysed as a continuous variable while gender, locality, province, educational level, and wealth index were analysed as categorical variables. Analyses were based on weighted data to account for the complexity of the survey design. Associations were analysed using Pearson’s chi-square test, and binary and multinominal logistic regression. Associations with a P = <0.05 were considered statistically significant.

The analysis was done using the Statistical Package for the Social Sciences for Windows Version 21.0 (International Business Machines Corporation-IBM Corp., Armonk, New York). The analysis aimed to study the association between dietary intake of processed foods and common chronic conditions. Cross-tabulation was done between the dependent variable (common chronic conditions) and all independent variables. To perform logistic regression the responses for all four food groups were converted into the dichotomous categories i.e. never consumed and ever consumed whereas the categories of the socio-demographic, behavioural, and economic variables were retained as available in the DHS.

Finally, multinominal logistic regression models were fitted to assess the association of processed food groups with the common chronic conditions, after adjusting for age (dichotomised as ≤36 or ≥37 years), gender (male or female) and wealth index as categorised in Table 1. As the model was only adjusted for the three mentioned confounding factors, it is possible that other factors were not considered due to lack of data, which may have resulted in residual confounding. The variables that demonstrated statistical significance in the cross-tabulations were selectively incorporated into the multinomial model. Also, as the total energy consumption data was not available from the dataset, it could not be integrated into the model.

**Table 1.** Socio-demographic characteristics of survey respondents (N = 10336)

Variable	Level	Frequency (n)	Percentage (%)
Region	Western Cape	754	7.3
	Eastern Cape	1352	13.1
	Northern Cape	882	8.5
	Free State	1031	10.0
	Kwazulu-Natal	1571	15.2
	North West	1085	10.5
	Gauteng	1031	10.0
	Mpumalanga	1220	11.8
	Limpopo	1410	13.6
Place of residence	Urban	5685	55.0
	Rural	4651	45.0
Ethnicity	Black African	8752	84.7
	White	451	4.4
	Coloured	986	9.5
	Indian/Asian/Other	147	1.4
Gender	Female	6126	59.3
	Male	4210	40.7
Educational level	No education	875	8.5
	Primary	1839	17.8
	Secondary	6668	64.5
	Higher	954	9.2
Current marital status	Never married	5369	51.9
	Married/Living together	3681	35.7
	Widowed/Divorced/Separated	1286	12.4
Wealth index	Poorest	2098	20.3
	Poorer	2227	21.5
	Middle	2337	22.6
	Richer	2066	20.0
	Richest	1608	15.6

## Results

The distribution of socio-demographic characteristics of the survey respondents is presented in Table 1. A total of 10,336 respondents, were interviewed from all nine provinces of South Africa. About 55% of the respondents lived in urban areas. Black Africans constituted 85% of respondents, followed by the coloured population, which accounted for about 10% of respondents. Of the total respondents, about 60% were women. Our analysis showed high educational attainment in South Africa. About two-thirds (65%) of the respondents had completed secondary education. More than half of the participants (52%) had never been married during the survey period. The mean age (in years) of the respondents was 39.32±18.2, which ranged from 15 to 95 years.

**Fig. 1.**
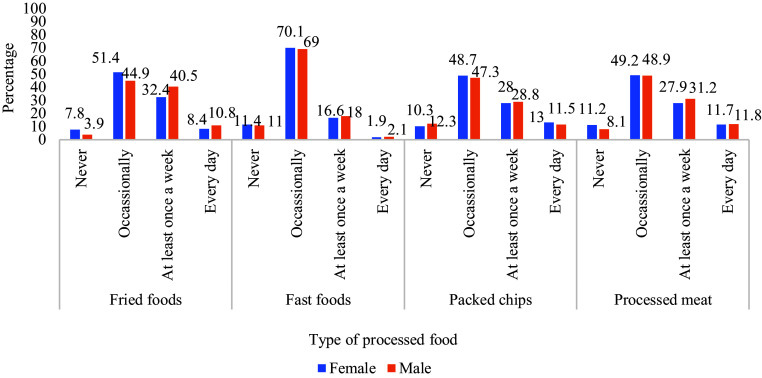
Gender-wise distribution of processed food consumption among survey respondents (N = 10336).

As shown in Fig. 1, about 10% of the male respondents ate fried foods daily, and 40% consumed them at least once a week (but not daily), which was higher as compared to female respondents (8% and 32% respectively). About 2% of male respondents ate fast food daily, and 18% did so at least once a week (but not daily). Thirteen per cent of the female respondents ate salty snacks (packed chips) daily, with 28% consuming them at least once a week (but not daily). Similarly, about 12% of male respondents consumed processed meat daily, and 29% consumed them at least once a week (but not daily). Overall, the consumption of processed foods was higher in male as compared to female respondents.

**Fig. 2.**
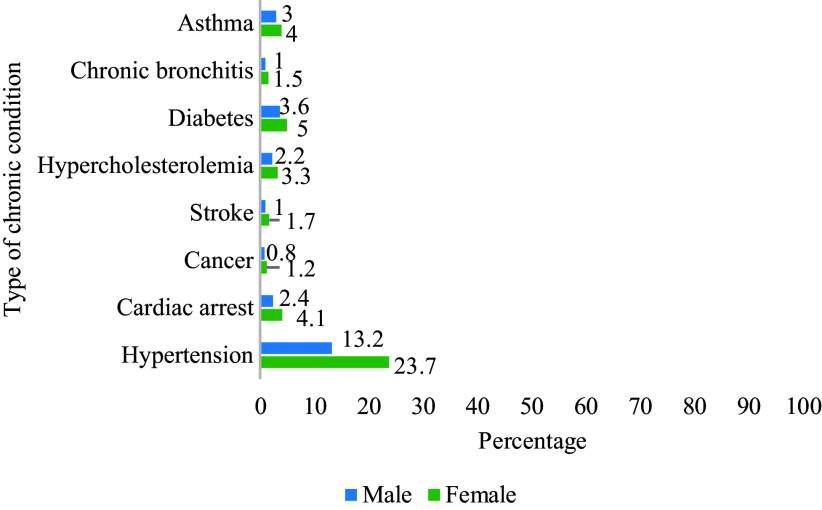
Gender-wise self-reported prevalence of common chronic conditions (N = 10336).

To ascertain the prevalence of the eight chronic conditions, respondents were asked if a doctor, nurse, or health worker had informed them of any of the chronic conditions (Fig. 2). High blood pressure was the most prevalent chronic condition reported among both female and male respondents (23% and 13%, respectively), followed by diabetes (5% and 3%). Cancer was the least common chronic condition reported among both female and male respondents (1% and 0.8%, respectively). Other chronic conditions were reported by 4 or fewer per cent of respondents. In general, the self-reported prevalence of chronic conditions was higher in female respondents as compared to male respondents.

Table 2 shows the logistic regression results of processed food groups and their association with eight chronic conditions.

**Table 2.** Regression results for consumption of processed food groups (ever consumed) against self-reported history of eight chronic conditions (N = 10336)

Variables	Crude Odds Ratio (COR)	95% CI		Adjusted Odds Ratio (AOR)	95% CI	
		Lower	Upper		Lower	Upper
**Hypertension**						
Fried foods						
No						
Yes	1.85^a^	1.55	2.21	1.08	0.88	1.34
Takeaway/fast foods						
No						
Yes	0.51^a^	0.45	0.59	1.42^a^	1.20	1.67
Salty snacks/packed chips						
No						
Yes	0.49^a^	0.43	0.56	1.56^a^	1.33	1.83
Processed meat						
No						
Yes	0.52^a^	0.45	0.61	1.34^b^	1.13	1.58
**Cardiac arrest**						
Fried foods						
No						
Yes	1.41	0.96	2.07	0.68	0.43	1.07
Takeaway/fast foods						
No						
Yes	0.52^a^	0.39	0.68	1.41^c^	1.00	1.97
Salty snacks/packed chips						
No						
Yes	0.43^a^	0.33	0.56	1.91^a^	1.40	2.60
Processed meat						
No						
Yes	0.48^a^	0.37	0.64	1.49^c^	1.07	2.08
**Cancer**						
Fried foods						
No						
Yes	2.37^b^	1.34	4.19	1.67	0.82	3.38
Takeaway/fast foods						
No						
Yes	0.50^b^	0.30	0.80	1.47	0.78	2.74
Salty snacks/packed chips						
No						
Yes	0.63	0.38	1.07	0.97	0.51	1.85
Processed meat						
No						
Yes	0.52^c^	0.31	0.87	1.36	0.73	2.55
**Stroke**						
Fried foods						
No						
Yes	2.44^a^	1.51	3.93	1.09	0.61	1.96
Takeaway/fast foods						
No						
Yes	0.33^a^	0.22	0.47	2.26^b^	1.41	3.63
Salty snacks/packed chips						
No						
Yes	0.49^b^	0.33	0.75	1.01	0.61	1.69
Processed meat						
No						
Yes	0.35^a^	0.24	0.52	1.84^c^	1.14	2.97
**Hypercholesterolaemia**						
Fried foods						
No						
Yes	1.41	0.93	2.14	0.94	0.57	1.54
Takeaway/fast foods						
No						
Yes	0.74	0.53	1.03	0.89	0.59	1.35
Salty snacks/packed chips						
No						
Yes	0.67^c^	0.48	0.92	1.09	0.75	1.60
Processed meat						
No						
Yes	0.42^a^	0.31	0.56	2.45^a^	1.74	3.45
**Diabetes**						
Fried foods						
No						
Yes	2.60^a^	1.97	3.43	1.60^b^	1.14	2.26
Takeaway/fast foods						
No						
Yes	0.44^a^	0.35	0.55	1.57^b^	1.16	2.11
Salty snacks/packed chips						
No						
Yes	0.48^a^	0.38	0.60	1.40^c^	1.05	1.88
Processed meat						
No						
Yes	0.53^a^	0.41	0.68	1.13	0.83	1.54
**Chronic bronchitis**						
Fried foods						
No						
Yes	2.26^b^	1.35	3.79	0.98	0.52	1.85
Takeaway/fast foods						
No						
Yes	0.44^a^	0.29	0.67	1.14	0.66	1.96
Salty snacks/packed chips						
No						
Yes	0.35^a^	0.23	0.52	1.85^c^	1.13	3.00
Processed meat						
No						
Yes	0.29^a^	0.20	0.44	2.44^a^	1.50	3.96
**Asthma**						
Fried foods						
No						
Yes	1.39	0.95	2.03	1.02	0.65	1.59
Takeaway/fast foods						
No						
Yes	0.77	0.57	1.04	0.95	0.65	1.37
Salty snacks/packed chips						
No						
Yes	0.58^a^	0.44	0.77	1.56^b^	1.13	2.16
Processed meat						
No						
Yes	0.63^b^	0.47	0.85	1.33	0.94	1.88

Level of significance:

a P < 0.001.

b P < 0.01.

c P < 0.05.

### Hypertension

After adjustment, three food groups i.e. takeaway/fast foods (AOR = 1.414, CI 1.197, 1.672), salty snacks/packed chips (AOR = 1.564, CI 1.334, 1.835) and processed meats (AOR = 1.337, CI 1.129, 1.583) showed increased odds of association with hypertension.

### Cardiac arrest

Like hypertension, after adjustment, three food groups i.e. takeaway/fast foods (AOR = 1.406, CI 1.002, 1.972), salty snacks/packed chips (AOR = 1.908, CI 1.400, 2.601), and processed meats (AOR = 1.497, CI 1.073, 2.088) showed increased odds of association with cardiac arrest.

### Cancer

In the binary logistic regression model, except for fried foods (COR = 2.375, CI 1.345, 4.195), other two food groups viz. takeaway/fast foods (COR = 0.496, CI 0.306, 0.804), and processed meats (COR = 0.522, CI 0.313, 0.871) showed lower odds of association with cancer. None of the processed food groups remained significant after adjustment.

### Stroke

When adjusted, of the four processed food groups, only two i.e. takeaway/fast foods (AOR = 2.265, CI 1.413, 3.632), and processed meats (AOR = 1.838, CI 1.138, 2.971) showed increased odds of association with stroke.

### Hypercholesterolaemia

After adjustment only processed meats (AOR = 2.451, CI 1.738, 3.457) showed higher odds of association with hypercholesterolaemia.

### Diabetes

When tested in the multinomial regression model, of the four processed food groups, two i.e. takeaway/fast foods (AOR = 1.568, CI 1.163, 2.114), and salty snacks/packed chips (AOR = 1.404, CI 1.049, 1.879) showed increased odds of association with diabetes.

### Chronic bronchitis

After adjustment, only two processed food groups i.e. salty snacks/packed chips (AOR = 1.846, CI 1.133, 3.008), and processed meats (AOR = 2.441, CI 1.506, 3.958) showed increased odds of association with chronic bronchitis.

### Asthma

In the multinomial regression model, only salty snacks/packed chips (AOR = 1.560, CI 1.127, 2.160) showed higher odds of association with asthma.

As per the logistic regression results following adjustment, at least three processed food groups were significantly associated with hypertension, cardiac arrest, and diabetes. Two processed food groups were significantly associated with stroke, and chronic bronchitis; one processed food group was associated with hypercholesterolaemia and asthma; and cancer was not associated with any food group. Processed meat and salted snacks/packed chips were each associated with five chronic conditions.

## Discussion

Urbanisation in developing countries fuels rapid changes in lifestyles that increase the risk of non-communicable diseases. Our analysis of DHS VII, South Africa showed high blood pressure and diabetes as the highest prevalent chronic conditions in both men and women. The analysis further reveals the association between processed food intake with seven out of the eight NCDs considered. Among the processed foods studied three showed significant association with hypertension, cardiac arrest, and diabetes. Two processed food groups showed significant positive association with stroke, and chronic bronchitis; one with hypercholesterolaemia and asthma; and cancer was not associated with any food groups. Processed meat and salted snacks/packed chips were each associated with five chronic conditions.

Pertaining to disease burden cardiovascular manifestations predominated in the South African region as per the analysis. Published literature from different regions in Africa has previously indicated the associated risk between overweight and obesity and intake of processed foods.^(8,21,22)^ Over the past two decades, an increased prevalence of overweight and obesity among adults and children has been documented in the South African region. Among the lifestyle factors, increased access to ready-to-eat foods have been identified as the leading cause of such trends.^(5,11,23)^ Our work specifically contributes to the scarce evidence between processed foods and their association with NCDs. The results highlight the shift from traditional diets resulting in low intake of fruits and vegetables, and essential fats proven to improve the health of populations.

Gender differences in dietary patterns are marked and vary across the globe. In our analysis dietary changes between genders identified a higher intake of processed food intake among men. Another analysis of the Ghana sub-national survey revealed contradictory observations, where women made unhealthy dietary choices, and were physically inactive.^(24)^ Similar interpretations were observed where, post-COVID, women displayed altered shopping patterns and consumed unhealthy foods.^(25)^ Few studies showed an increased risk of overweight and obesity in women with increased intake of processed foods.^(21)^ However, the GBD analysis did not find gender differences in the prevalence of cardiovascular events in the Sub-Saharan region. Both men and women reflected higher DALY rates for NCDs compared to the global average.^(26)^

Hypertension and processed foods: The findings showed a significant association between takeaway/fast foods, salty snacks/packed chips, processed meat, and hypertension and cardiac arrest. Few studies have demonstrated a positive association between processed foods and cardiovascular health in the African continent that aligned with our findings.^(27,28)^ Sodium along with other additives such as spices, sulphites, and preservatives in processed foods increased their palatability and shelf-life. This combined with packaging and marketing increased purchases and consumption.^(29)^ To reduce the risk of NCDs, South Africa was the first country to regulate mandatory salt reduction in processed foods.^(30,31)^ Although regulations on individual nutrients are likely to benefit, those that facilitate the production and distribution of healthy foods would be a major step towards a healthy food environment.

Stroke and processed foods: The association between stroke and takeaway/fast food, and processed meat intake were significant in our analysis. Processed food groups that are not plant-based, but energy-dense, combined with physical inactivity have been shown to increase the risk of stroke in other studies.^(32,33)^ High intake of sweets, fried foods, sugar-sweetened beverages, processed meat, and low intake of fruits in the African diet have previously shown significant associated with stroke.^(34,35)^ Although the case-control study that aimed to quantify country-specific risk factors failed to identify any dietary behaviours for the African continent, it provided further evidence of the link between processed foods and the risk of stroke. Further, hypertension has emerged as a modifiable risk factor to prevent stroke.^(36)^ Increasing evidence thus points to the consumption of processed foods and its associated risk with multiple chronic conditions.

Hypercholesterolaemia and processed foods: A high intake of salty snacks/packed chips and processed meat was associated with hypercholesterolaemia, in our analysis. A systematic review of studies in Africa highlighted that 80% of dyslipidaemia was associated with diet and lifestyle factors.^(37)^ Similar work from Nigeria recognised high fat intake and unhealthy lifestyles as determinants of hypercholesterolaemia.^(38)^ Evidence from a cohort study presented hypercholesterolaemia as a co-morbidity with other metabolic conditions.^(39)^ Despite less evidence in the association with specific plant-based diets, populations have been encouraged to consume traditional fruit and fibre-rich plant-based diets, to prevent metabolic conditions. More research is warranted to study diet and dyslipidaemia in Africa.

Diabetes and processed foods: Our work identified intake of takeaway/fast foods and salty snacks/packed chips specifically associated with increased risk of diabetes. Increased consumption of processed and ultra-processed foods has been linked with diabetes in Africa.^(11,40)^ Women who purchased food frequently from outside the home showed a higher risk of NCDs including diabetes.^(41)^ In contrast, severe food insecurity showed an association with diabetes in Angola, a characteristic feature of the double burden in developing countries.^(42,43)^ Food insecurity among the lower socio-economic populations exposed them to low-cost processed foods that are likely to increase the risk of NCDs. Developing countries face the dual challenge of addressing food insecurity and promoting healthy diets to prevent NCD risks among various economic subgroups in the population.

Non-metabolic conditions such as asthma and bronchitis and their association with processed foods: Salty snacks/packed chips and processed meat intake were significantly associated with respiratory tract conditions in our analysis. The association between ultra-processed food intake and respiratory tract conditions has been documented widely. Work among the Spanish population showed a significant association between the intake of processed foods and asthma and bronchitis.^(44)^ When the inflammatory potential of diets was tested, processed foods showed a high potential for inflammation.^(29,45)^ The GBD study on the dietary risk factors that impact health in 195 countries (2019) showed exposure to processed foods with high sodium intake and low intake of healthy foods as leading dietary causes of diseases in these countries.^(2)^

Limitations: Our systematic analysis of the DHS VII data had several limitations. The analysis used secondary data and the indicators of dietary behaviour are limited to processed meats, packed chips, fast foods, and fried foods. Other unfavourable dietary behaviours such as the frequency of eating out or foods from the NOVA classification (it groups foods according to the nature, extent and purpose of the industrial processing they undergo) such as sugar-sweetened beverages or other energy-dense desserts have not been included in the DHS 7 data. This possibly limited the scope of our analysis. We also did not study the associations between the intake of processed foods and undernutrition. The strength of the association also varied between the different foods studied and the NCDs. Considering the complexity of the data, our analysis also did not consider the frequency of processed food consumption and the risk of NCDs. Also, in this study, numerous associations between several processed foods and several NCDs were explored without strong a priori hypotheses. This approach may lead to chance statistical findings. Therefore, it is important to consider the limitations of this study and to interpret the results with caution. Additionally, further research is needed to better understand the associations identified in this study.

An analysis of nationally representative data to study associations between diet, and disease relationships are likely to be affected by a comprehensive list of confounding variables such as lifestyle or socio-demographic factors. This could explain the observation in our results, of the 15 combinations of food exposure and disease outcome where both the crude and the adjusted odds ratio were statistically significant, 14 showed a change in the direction of the association from a negative crude odds ratio to a positive adjusted odds ratio. This is suggestive of strong confounding by one or more of the covariates which is beyond the scope of this analysis. Other limitations related to measurements were self-reported NCDs, absence of reference period for food consumption, nutrient adequacy using a 24-hour diet recall, and diet diversity.

In summary, we found that the consumption of any of the processed food groups increased the risk of NCDs in the South African population. Enabling policy and regulatory efforts in the production and distribution of processed foods, combined with improved awareness among the population need to be prioritised for immediate action. Access to processed foods should be limited, to minimise exposure. Healthy alternatives to improve the diets should be prioritised in health communication. Awareness of natural and minimally processed foods would enable healthy dietary choices. Facilitating the populations to choose traditional healthy diets would be a sustainable strategy for the prevention of NCDs.
